# Faceting the post-disaster built heritage reconstruction process within the digital twin framework for Notre-Dame de Paris

**DOI:** 10.1038/s41598-023-32504-9

**Published:** 2023-04-12

**Authors:** Antoine Gros, Anaïs Guillem, Livio De Luca, Élise Baillieul, Benoit Duvocelle, Olivier Malavergne, Lise Leroux, Thierry Zimmer

**Affiliations:** 1 UMR CNRS/MC 3495 MAP, Modèles et Simulations pour l’Architecture et le Patrimoine, 13402 Marseille, France; 2 Laboratoire de Recherche des Monuments Historiques, 77420 Champs-sur-Marne, France; 3 UMR CNRS/Univ. Lille 8529 IRHiS, Institut de Recherches Historiques du Septentrion, 59000 Lille, France; 4 SJTU Paris Elite Institute of Technology, Shanghai, 200240 China

**Keywords:** Engineering, Information technology, Applied mathematics

## Abstract

April 15th, 2019: Notre-Dame Cathedral in Paris was burning, the spire collapsed on the nave, vaults crumbled and most of the timber roof was gone. In the post-disaster context, the authenticity and the monitoring of the archaeological remains are crucial for their potential reuse during reconstruction. This paper analyzes the collapsed transverse arch from the nave of Notre-Dame as a case study of reconstruction, using the digital twin framework. We propose four facets for the digital twin experiment—physical anastylosis, reverse engineering, spatio-temporal tracking of assets, and operational research—that are described in detail, while being assembled to support a hybrid reconstruction hypothesis. The digital twin can realize the parallel unfolding of physical-native and digital-native processes, while acquiring and storing heterogeneous information as semantically structured data. The results demonstrate that the proposed modeling method facilitates the formalization and validation of the reconstruction problem and increases solutions performances. As result, we present a digital twin framework application ranging from acquisition to data processing that informs a successful hybrid reconstruction hypothesis.

## Introduction

### A scientific action for the restoration of Notre-Dame

**Figure 1:**
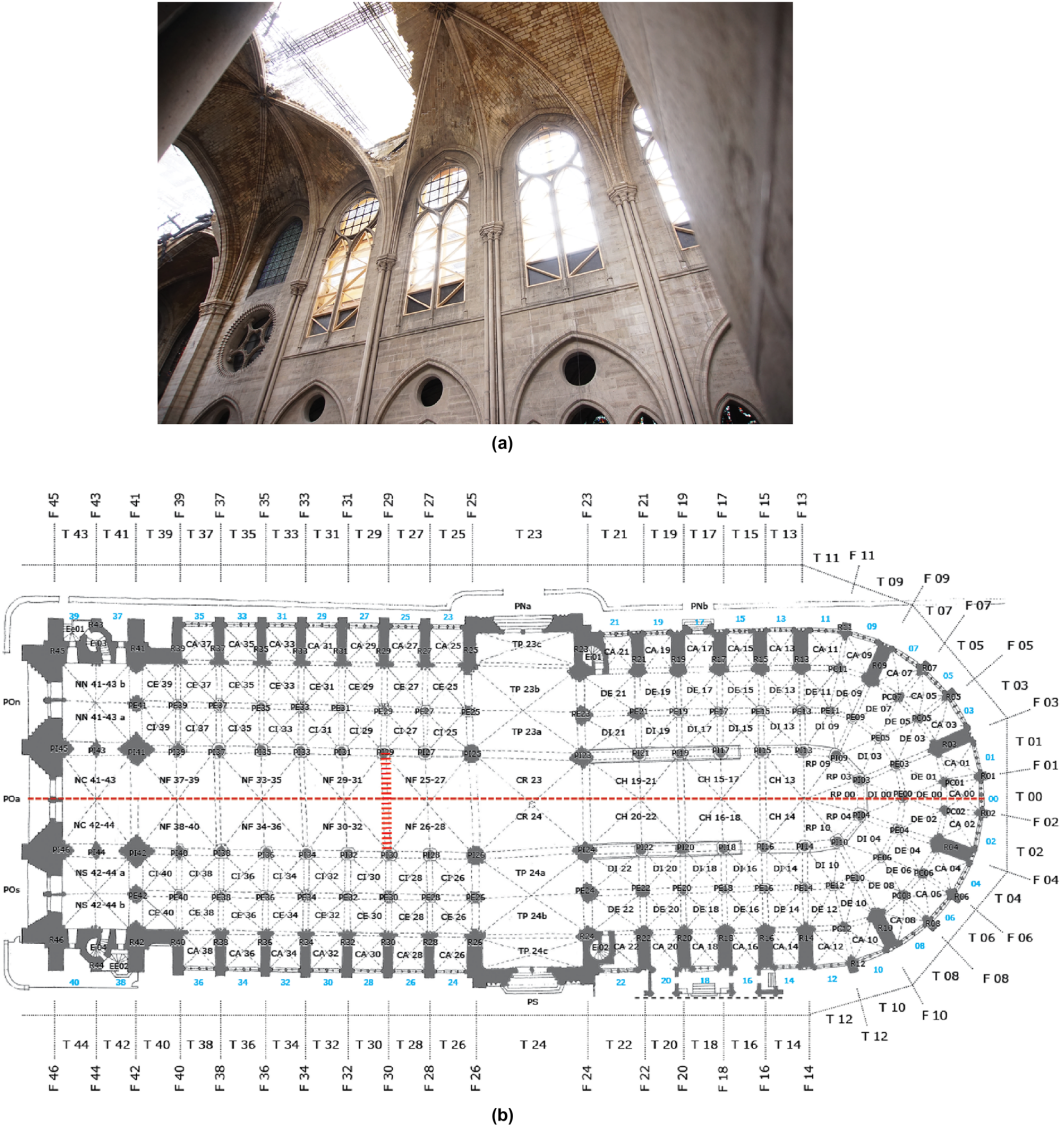
(**a**) Collapsed vaults in the nave and the transept of Notre-Dame (@Bestrema); (**b**) Situation of the collapsed nave vault on the floor plan (@RNDP).

**Figure 2:**
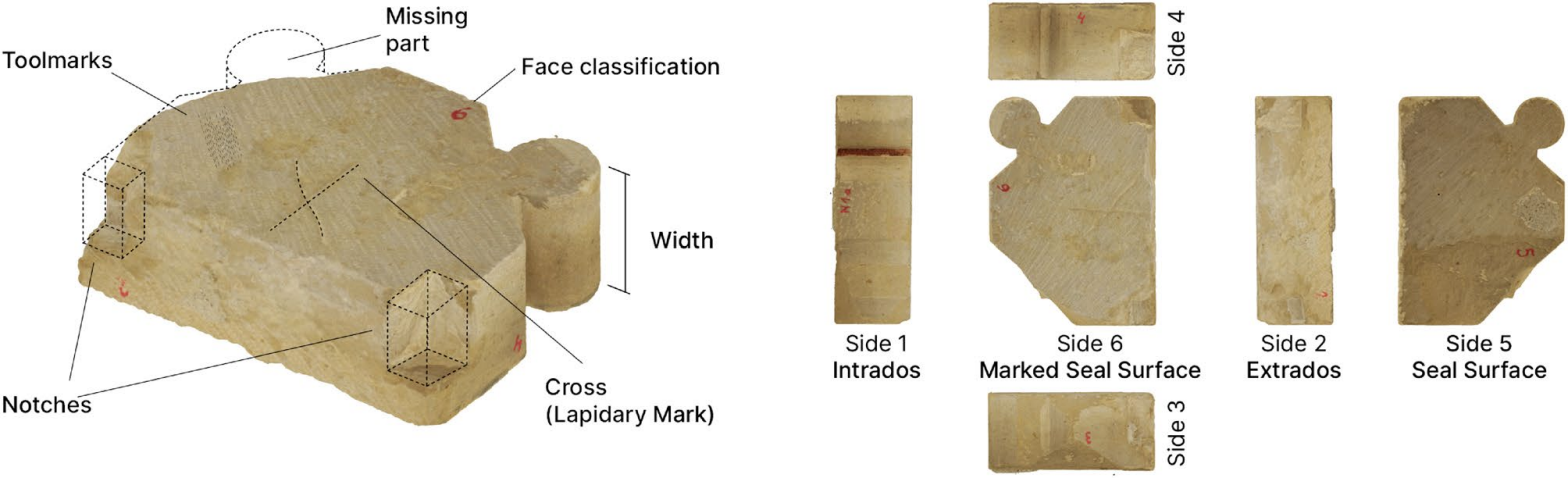
The components of the collapsed arch: the voussoirs, standardized and manufactured building elements.

April 15th, 2019: Notre-Dame Cathedral in Paris was burning, the spire collapsed on the last two spans of the nave and most of the timber roof was gone above the vaulted ceilings (Fig. 1). The day after the Notre-Dame fire, the rebuilding work of the cathedral was organized separately from the scientific research on Notre-Dame: at stake was the gathering of new data in the field while allowing the work of rebuilding and restoration to be done in the shortest time frame. More than 170 scholars are involved today in the scientific research on Notre-Dame divided by working groups focused on Stone, Metal, Timber, Stained Glass, Civil Engineering, Acoustics, Sculpture and Ornamentation, Anthropology, and Digital Data. It represents an unprecedented scientific effort to study the monument^1^. Post catastrophe, the first priority was given to the safety measures of strengthening and scaffolding works for the stabilization of the monument, followed by the removal of remains, cleaning and depolluting activities before addressing design planning. The scientists of the Historical Monuments Research Laboratory (LRMH) and state archaeologists played a leading role in the sorting of material remains for the initial inventorying and emergency documentation protocols^2^ to document the stone remains, followed afterwards by the Stone working group. By highlighting the potential and the limits of a digital twin driven approach within the framework of the architectural heritage restoration, this paper documents the results of a collaborative experiment between the Digital Data and Stone working groups to address the reconstruction of a collapsed part of the nave vaulted ceiling to the pre-disaster state. The uniqueness of the reconstruction question of the Notre-Dame transverse arch lies in the particular case where most of the voussoir elements of the transverse arch are available for study. Surprisingly enough, most of them were not destroyed during the fire or their fall. Hence, if the reconstruction can be compared to a jigsaw puzzle, in this case, only few puzzle pieces are missing. From the total of 79 fallen voussoir elements, 76 are identified from the remains, and 71 have been analyzed at the time of this paper’s publication (Fig. 2).

The formulation of a faithful pre-disaster state hypothesis is the main objective in cultural heritage reconstruction projects, as authenticity is “the essential qualifying factor concerning values”^3^. A primary characteristic of the Gothic building-style era is the sexpartite vaulted ceiling with the transverse pointed arches as main enablers of mechanical stability and lightness for the covering. Notre-Dame’s vaulted ceiling bears witness to a technical milestone due to the height they reach, introducing large windows in the walls and filling the nave with light. The London Charter^4^ advocates for transparency in the use of 3D models in cultural heritage, while the Principles of the Seville Charter^5^ proposes implementation criteria for the London Charter on the use of 3D models for virtual reconstructions. Following these recommendation documents, our reconstruction study can then be defined as a virtual anastylosis as the “restructuring existing but dismembered part in a virtual model” or a virtual reconstruction as the “use of a virtual model to visually recover a building or object made by humans at a given moment in the past from available evidence of these buildings or objects, scientifically comparative inferences”^5^. But these definitions do not cover the totality of our research question. The reconstruction question in Notre-Dame does not fall within the typical case of virtual reconstruction study, where the built asset is usually documented in its decayed condition and the reconstruction hypothesis partially overlaps with the physical object. Here the virtual reconstruction intertwines with the restoration project of the physical monument, as it is documented (surveys, photos collections etc.) of the monument itself (building) and its components (photogrammetric data acquisition of voussoirs). This peculiar operational context brings us to consider the alternative method of digital twin to explore the question of reconstruction and build these mandatory connections between the digital and physical assets and their interactions.

### A digital ecosystem accompanying the scientific research

Since the beginning of the scientific action on Notre-Dame^6^, the Digital Data working group^7^ has been working towards an innovative digital ecosystem to accompany the scientific research and the restoration of the cathedral by progressively integrating the data coming from the actors involved in the knowledge production of current state and previous states of the cathedral (historians, archaeologists, architects, engineers, physicists, chemists, etc.), as well as the actors dealing with the restoration of the cathedral (project owner, project managers, curators, conservators, etc.). The digital ecosystem offers coherence for digital native processes but the duality physical object/digital object remains an obstacle in building data-driven approaches for hybrid-processes.

### The digital twin experiment for reconstructing a collapsed arch

**Figure 3:**
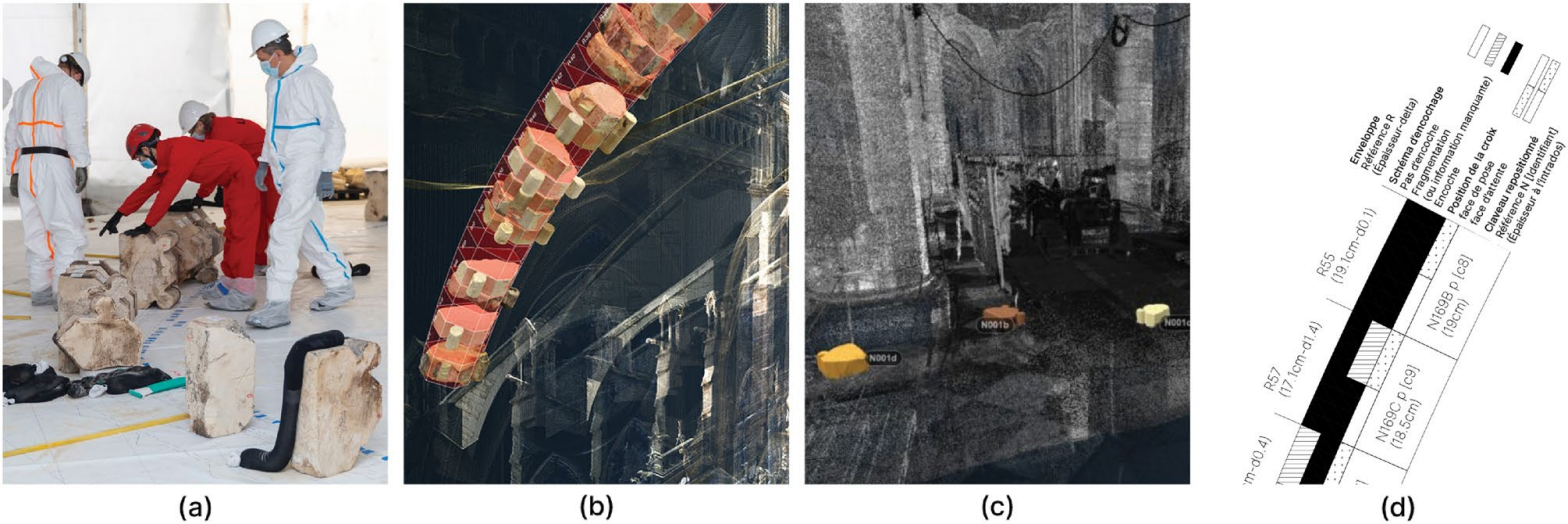
Assembly of four processes for the reconstruction: (**a**) physical anastylosis, (**b**) reverse-engineering hypothesis, (**c**) image-based spatio-temporal annotation and identification of voussoirs, and (**d**) operational research.

**Figure 4:**
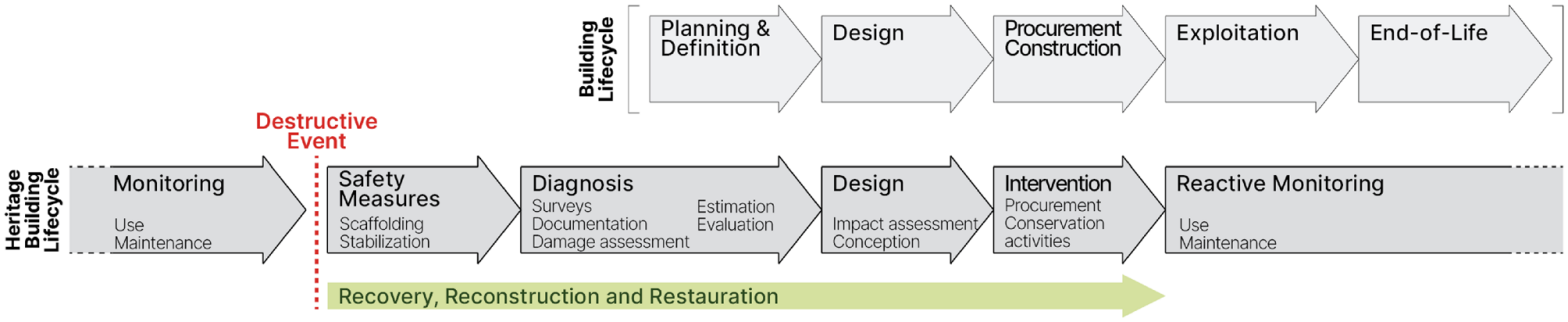
Building life cycle and Notre-Dame restoration project as continuous cultural heritage building life cycle.

To overcome the aforementioned challenges, we designed and experimented with a novel kind of digital twin for post-disaster heritage building reconstruction on the collapsed transverse arch of Notre-Dame de Paris cathedral, more specifically, the voussoirs (*in situ* and *ex situ* remains), the cathedral under restoration, the voussoirs to be cut, and the rebuilt arch. It is conceived as a thorough process-driven experimentation of the digital twin framework, rooted in the existing digital ecosystem. The digital twin has progressively been built following the development of the expert processes for the reconstruction in 4 methodological facets: physical anastylosis, reverse engineering, spatio-temporal annotation of remains, and operational research (Fig. 3). In the course of the cultural heritage building full lifecycle (Fig. 4), these facets emerged and were assembled^8^, the links between the physical and digital assets have been created at every step to strengthen the digital twin consistency. The knowledge continuity is sustained over the lifecycle with a graph database driven by a core ontology and pattern oriented.

Below, we outline the methods that we used to address the reconstruction question: the heritage digital twin application experiment allows us to mimic the design of the voussoir components and of the building, as well as their interaction in the digital realm (section “Iterative digital twin modeling in cultural heritage in unpredictable context”). Our use of digital twin in heritage is based on the need for modeling the semantic links and for articulating data from the 4 complementary facets of the reconstruction process (Fig. 3): namingly, the physical anastylosis (section “Physical anastylosis”), the reverse engineering (“Reverse engineering”), the spatio-temporal tracking of the voussoirs position (section “Spatio-temporal annotation and identification of archeological remains”), and the operational research model (section “Operational research”). Although the spatio-temporal annotation and operational research are a novelty for addressing the study of reconstruction, none of these four methods was sufficient by itself to provide a suitable reconstruction hypothesis. But once articulated and enriched by one another’s results, the hybrid reconstruction hypothesis provides a successful solution. This hybrid hypothesis is only possible thanks to the knowledge continuity in the digital twin application framework (section “Knowledge continuity, data modeling and management”). Then we will then present the results of the digital twin experimentation and the hybrid hypothesis reconstruction (section “From physical anastylosis empirical predicates to operational research decision making”). We present the methodological reconstruction facets of the digital twin experiment through the operational research lense and propose an analysis of the final results. We conclude with a discussion on the use of digital twins for case studies in cultural heritage (sections “Discussion” and “Conclusion”). Although our digital twin is specific to our reconstruction research question in Notre-Dame, we discuss the potential use of digital twin application for building knowledge in heritage science.

## Methods

### Iterative digital twin modeling in cultural heritage in unpredictable context

Why using digital twin technology for post-disaster reconstruction of a cultural heritage building? Digital twin is employed for complex system modeling with a tailored feedback between physical and its twinned digital asset, specific to a process, and the links engineered between them. Originally designed for the aerospace industry^9^, it finds a natural extension to manufacturing^10^, and then to construction^11^. A nascent trend arises on the topic of the heritage digital twin^12,13^ in extension to Heritage BIM (HBIM) research^14,15^. But this trend does not allow yet the foundation for the use of digital twins in production for heritage context alongside the full building life cycle. As a foundation for our research, digital twin for manufacturing^10,16^ and for construction^11,17^ effectively describes the application framework for binding physical and digital counterparts by a connection for unique, complex and costly assets^18^. Despite the difference in the context of application, the following definition of digital twin suits well to heritage monuments: “a set of virtual information constructs that mimics the structure, context and behaviour of an individual or unique physical asset, that is dynamically updated with data from its physical twin throughout its life-cycle, and that ultimately informs decisions that realize value”^19^. The digital twin technology offers a framework to consider the value and resilience of heritage assets from their construction to their (partial) destruction and restoration (Fig. 4).

The collapsed nave arch of Notre-Dame is a complex and unique physical built asset which condition goes through different states and needs documentation in its full building life cycle (Fig. 4): (a) *in situ* transverse arch before fire, (b) as collapsed arch and rib stone remains, (c) reconstruction hypotheses of the arch, (d) stone cutting for rebuilding, and (e) sensor structural monitored arch. The cultural heritage building life cycle is roughly equivalent to the full building construction life cycle for design and construction phases, but there are some major differences. First, the heritage building life cycle starts with phases of safety measures implementation and then mandatory diagnosis. The post-disaster safety measures are quick actions to reinforce the building. They prevent the building from further damage and allow people to enter the building and start working safely. These emergency measures are only temporary, while the diagnosis completes the damage assessment, surveying, documentation gathering, authenticity assessment, and strengthening planning. The second main difference is the stress put on monitoring and maintaining on a permanent basis, as stated by cultural heritage international policies^20^ “to maintain their authenticity and integrity”^21^. In other words, when an asset is assessed to have some heritage values, it often starts a process of preemptive endless preservation. The corollary is the theoretical absence of end-of-life for these cultural heritage assets that are then continuously maintained and monitored.

**Figure 5:**
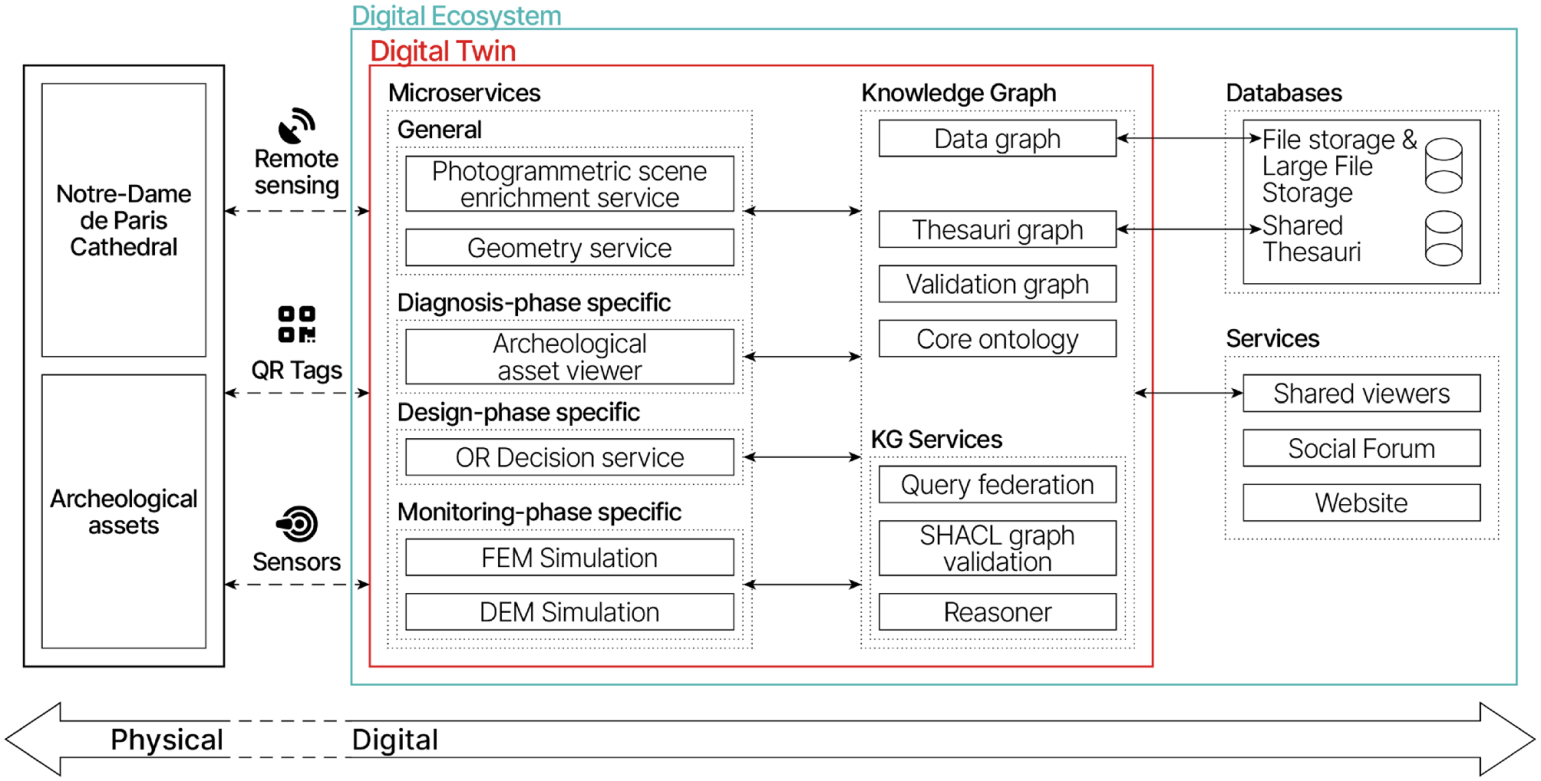
High level overview of the digital twin model composition and its links to the existing digital context.

These differences with the typical building life cycle impact the modeling of our cultural heritage digital twin (Fig. 5). We advocate for an iterative modeling method, rooting the reconstruction question as a part of the design phase. On one hand, new links are to be designed to maintain or make the key physical/digital connection. The strategy is an opportunistic one: strengthen the connection by stacking the links iteratively along the phases. Following the state-of-the-art choice for cultural heritage building digitization and monitoring, remote sensing via both TLS and photogrammetric surveys produces the compulsory geometric representation layer of the built asset and its disbanded parts. These operations are performed either manually, as the pre-disaster TLS point cloud model acquired in 2010 by A. Tallon^22^(average density of 0.8 pt/mm) or automated for survey coherence among similar assets, such as the machine-assisted photogrammetric survey carried on the voussoirs (320 photos, average density of 2pt/mm). In the diagnosis phase, more precisely during the lapidary study inventory^23^, the need to go back and forth between the physical asset and its digital surrogate pushed for the use of QR code external tags for the voussoirs that complement the metadata links in the inventories. From the physical assets, the links reach the following digital elements: the TLS and photogrammetric cathedral surveys, voussoirs photogrammetric models, parametric digital model, operational research models, knowledge models, and simulation models.

On the other hand, from a computational perspective, the unpredictable event conservation activities and ongoing scholarly processes imply complex and messy computational workflows. We recognize this as one of the primary challenges for the cultural heritage digital twin modeling. In order to mitigate the complexity and messiness of processes, microservice architecture is an enabling paradigm based on a divide and conquer strategy allowing to stack *ad hoc* stateless and autonomous processes. As a Service Oriented Architecture, microservices are loosely coupled to provide the necessary flexibility while allowing a high degree of cohesion within phase-specific microservices and opportunistic cohesion with more broad services (such as geometry operations handling). Each microservice offers a REST interface and is capable of querying the SPARQL 1.1 Graph Store interface of the RDF knowledge graph (section “Knowledge continuity, data modeling and management”). In such a way, integration of new functionalities in the digital twin alongside project phases is eased, tasks can be allocated more clearly between the actors due to the autonomy of the microservices. In addition, seamless integration of the digital twin within the existing digital ecosystem is granted regardless of its architecture.

### Framing the facets of the hybrid hypothesis for the reconstruction

#### Physical anastylosis

The reconstruction question brings together the architects of the restoration, archaeologists, and heritage science researchers to uncover socio-cultural and technical aspects of the collapsed arch and test initial hypotheses. The reconstruction studies usually go hand in hand with reconstruction representations (drawings or 3D models) but the choice was made to test the reconstruction hypothesis at the scale 1:1 with a physical anastylosis, that is a test for reassembly of the arch from its pieces. The aim is twofold: locate the original position of the components with the support of a $15\times 10$ m tarpaulin printed with the arch layout extracted from Andrew Tallon’s point cloud. And gather archaeological and geometrical information on both the arch and its components. This step of physical anastylosis is a turning point in the work on the reconstruction question. It limits the mediating effect of the representation in the early stages of reasoning and stands out as a unique experience for an arch of these dimensions (Fig. 3a). These experiment results add up with the preliminary lapidary study practiced on the physical voussoirs, later on their twins^23^. From this experience, leads for the transverse arch reconstruction are identified (Fig. 7). An expert eye could observe that the geological facies of the limestones constituting these blocks show variations allowing the pairing of stone pieces (as voussoir series) that were cut in the same geological level^24^.

#### Reverse engineering

The physical anastylosis gave limited but key results due to the time and space constraints on site. At this point, it was necessary to reshape our approach to the question in order to carry on our investigation. Reverse engineering aims at “developing a set of specifications for a complete hardware system by an orderly examination of specimens of that system [...] without the benefit of any of the original drawings”. This set of specifications consists in the synthesis of both the dimensional specification (the part dimension and material) and the functional specification (the adjustment and tuning of product parts interaction during manufacturing from design recovery and testing)^25^. Accordingly, reverse engineering-based methodologies are extended to address the as-built building and cultural heritage site modeling challenges^26^. For instance, research developments concern scan-to-BIM with segmentation and classification using prior architectural knowledge for multimodal optimization^27^ or AI techniques instantiating idealized parametric primitives^28^. Hence the literature broadly focuses on dimensional specification to encompass digitization, feature extraction and CAD modeling of the parts in one automated process, over the functional specification.

In the reconstruction task at hand, we identify the transverse arch as a system through the reverse engineering lens. The transverse arch is a sub-assembly of the vaulted ceiling, composed of voussoirs, i.e. its interlinked components. The considered arch is dismantled but we dispose of 7 similar neighboring transverse arches in the Notre-Dame nave. In the case of cultural heritage buildings, we cannot dismantle other arches, only non-destructive techniques are advisable. Hence the dimensional specification of the transverse arch stems from remote sensing operations at different scales and levels of the system (section “Iterative digital twin modeling in cultural heritage in unpredictable context”). A geometric parametric model (Fig. 3b) is crafted to fit the pre-disaster Tallon’s point cloud. The objective is to extract dimensional design from the vaulted ceiling before its collapse. The parametrization is based on craftsmen design knowledge of arch drawing. The arch curvature measured on the pre-disaster point cloud is not constant. Therefore, we fit the construction lines of the parametrized arch voussoirs using nonlinear regression^29^. As output, the modeled voussoir volumes can be read as slot locations for the reconstruction task and visualizations in a shared digital environment 3D viewer (Fig. 3b). This step does not produce one representation of a given reconstruction hypothesis in 3D but rather represents the reconstruction problem itself with the parametrized voussoir location slots in 3D. It results in a shared digital resource to carry on the reconstruction discussions started with the physical anastylosis. In contradistinction, the functional specification started during the work sessions of the physical anastylosis. For the reverse-engineering analysis, the physical anastylosis can be understood as a design recovery process: it mobilizes domain knowledge and fuzzy reasoning to “identify meaningful higher level synthetic abstraction, beyond those obtained by examining the system itself”^30^. As result, the predicates, the assertions, the qualitative and quantitative characterization of the components from the physical anastylosis provides, once systematized, an initial functional specification of the reverse-engineered system (Fig. 7).

#### Spatio-temporal annotation and identification of archeological remains

**Figure 6:**
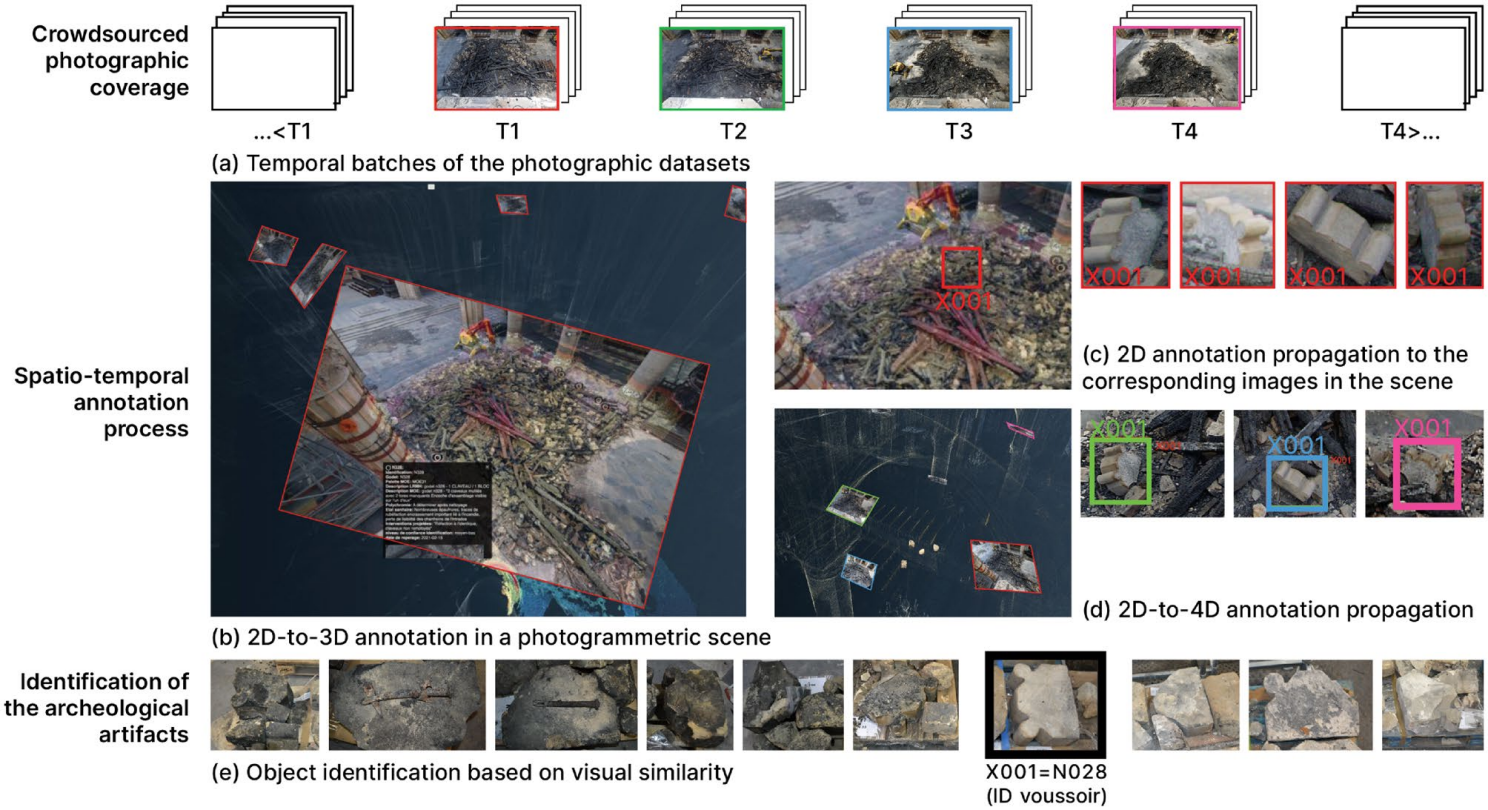
Spatio-temporal tracking in photogrammetric scenes, identification, and annotation of voussoirs in the remains during the recovery operations.

From the moment they fell to the end of the cleaning operations in the cathedral, we want to identify the path of the voussoirs in the remains up to the sorting and collecting operations for inventory and study. At every pass of the machine, voussoirs appear, get extracted, or get buried in the remains, and so on. The difficulty of this research is double: first, the voussoirs are only partially visible in the remains and mixed up with dust, ash, and other built material remains. Second, the archaeological artifacts move during the cleaning operations due to the use of unmanned machines, so their location is not stable in space and time. Using the crowdsourced photographic coverage from LRMH (13,000 images) made daily during the operations of removal of remains and cleaning after the fire, we apply a novel approach of spatio-temporal documentation using temporal photogrammetric annotation. The voussoir identification is sometimes possible thanks to the initial inventory, the voussoir collection, and the inventory photographs (Fig. 6). This method is based on the complementary use of image analysis and classification, image-based 3D geometric modeling, and semantic annotation techniques. The spatio-temporal retrieval method is structured in 4 steps. (a) The set of photographic images is segmented by temporal criterion into batches representative of the main stages of the recovery operations. (b) The batches are transformed into 3D photogrammetric scenes on the Aïoli platform^31,32^, 3D point cloud are associated with the spatialisation of multi-temporal 2D images. (c) The projective link between the 2D images and the 3D scene allows interactive 2D annotations on a single image to be propagated in the corresponding point cloud and the corresponding images where the selected element is visible. The geometric representation of an annotation is associated with semantic attributes defined by the user. (d) The spatial and semantic annotations relative to a temporal span are extended over other temporal spans thus the presence of the selected element is checked by comparing the point clouds of the 3D region and/or the 2D projections of the relative images.

It is then possible from a single annotation of a voussoir on a single image to automatically identify its position in the 3D scenes and in the corresponding 2D images in the pile of rubbles, (e) then to extrapolate the date and time of the last photograph representing it before its recovery and storage on pallets. All of this information is used to associate a voussoir found in the scene with a single voussoir preserved on the pallets.

#### Operational research

**Figure 7:**
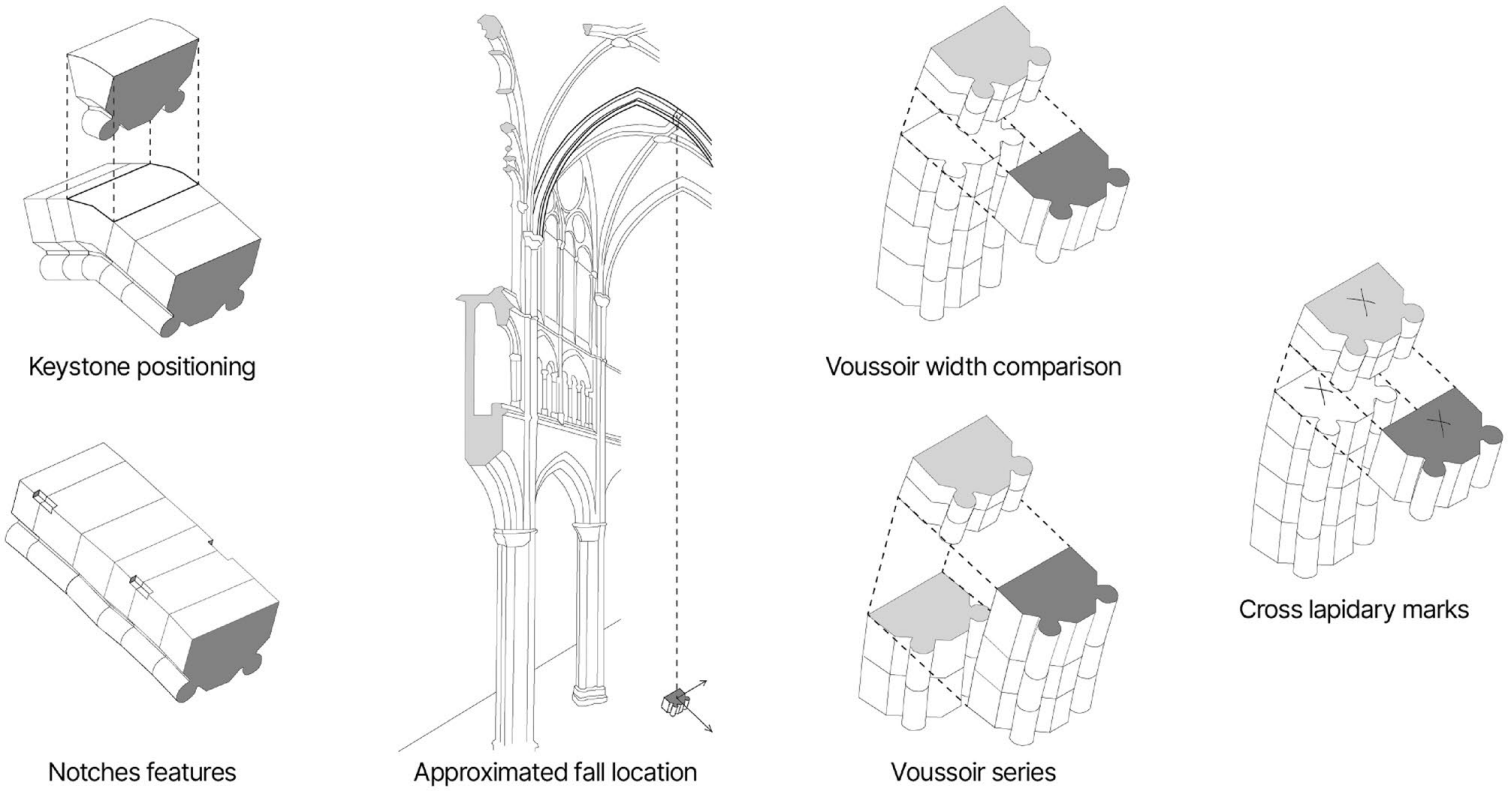
Set of the archeological predicates used in the reconstruction study.

Optimisation is a go-to technique to improve design performance on such criteria as structural or thermal, relying on simulation predictions to rightly manufacture and arrange the parts. As such, reconstruction is the special design case where parts pre-exists and must be returned as is to maximize the main performance value, that is authenticity (section “Iterative digital twin modeling in cultural heritage in unpredictable context”). This authenticity qualitative value is conversely substituted by the location assignment value—i.e. putting the right voussoir at the right slot—that is quantitative. In this research, we adapt the assignment problem^33^ to formulate the NP-Hard collapsed arch problem. The LP model objective function is formed from physical anastylosis predicates and reverse engineering measures (Fig. 7).

As explainability of the models and algorithms within the digital twin is a mandatory prerequisite in collaborative contexts, we reached the understanding of contributors through soft operational research facilitation of the problem structuring. Thus, shared understanding and commitment^34^ are built as the viewpoints of the participants are valued and recognized during debate and its elicitation. The outcomes are the quality and acceptance of the hybrid solutions among the contributors and stakeholders, and the traceability and pertinence evaluation of any systematized premice in the fields, seen as epistemic contexts. Therefore the reconstruction hypotheses were continuously reviewed and validated with the archaeologists, architects, material and conservation scientists, art historian and conservators. As it fits with the reconstruction overall question, we used the jigsaw-puzzle solving task as a driving metaphor for facilitation. From the operational research perspective, jigsaw-puzzle solving is a specification of the assignment problem^35^, sharing the same logical basis and performance indices—rightful location of matching elements—as the reconstruction problem. However, most of the literature focuses on shape and pictorial content matching^36^ while most reconstruction clues are rather of semantic nature^37^. The iterative modeling process allows us to follow the intermittent updates on data and predicate statements. Alongside with the iterations, a layered validation was applied to tackle the crucial validation point. We generate a reference system following the conceptualization of the problem, where predicates are held to be the system characteristics. Data is statistically deduced from physical anastylosis and reverse engineering observations and models, from their corresponding uncertainties. First layer is validation by construct^38^, a predictive validation where “the model is used to predict the [reference] system behavior” and compared “if they are the same”. Second layer is a sensitivity analysis—and model behavior exploration—carried out with degenerate tests and extreme condition tests on the reference system.

### Knowledge continuity, data modeling and management

The data management strategy for scientific information requires awareness on both the provenance and heterogeneity of data. Reproducibility, collaboration, and interoperability are at stake. Indeed, the status of the information—primary or secondary source, hypothesis, interpretation, measurement, simulation prediction, etc.—conditions the use of the data. Whereas the typical big data challenges push toward holism, data-driven digital twins entail efficiency in data processing and knowledge continuity. The semantic web stack provides a technological foundation identified as enabling digital twin knowledge graph applications^17^ using domain ontologies focused only on sensors^39^, testing process or cultural heritage^12,15^. However, domain ontologies and IFC/BIM based structures link expert knowledge and provide desired efficiency to a specific process, but due to their narrow applicability, a comprehensive digital twin deployment and iterative extension is hindered. While sharing the same technological basis, a solution for knowledge modeling in digital twins is to opt for a pattern-oriented data modeling method, built upon an ontology of a higher degree of abstraction, i.e. a core ontology. We outline the capacity of the latter to describe data provenance, physical and digital entities, and different granularities of spatio-temporal relations. We use the CIDOC CRM, a standard core ontology applicable for cultural heritage documentation^40^. Pattern making is a bottom-up modeling approach centered on the data and events, webbing a cohesive cluster of information around it and maintaining loose links with the existing clusters in the graph. Three mechanisms of extension allows flexibility and instantiation of the pattern as a data structure: (a) instantiation of the classes of the core ontology using a poly-hierarchy of types from a controlled vocabulary structured in SKOS; (b) opportunist insertion of domain ontology classes to specify the semantics of a data mapped to the core ontology; (c) ontological extensions and profile application such as CRMsci, CRMdig, CRMcr for CIDOC CRM. In order to assert the consistency of the knowledge graph, each pattern is paired with a validation graph with SHACL rules to be processed within a third party service (Fig. 5). In addition, these rules allow coherence among federated queries to be sent within the digital ecosystem to other knowledge graphs, whether native triples or dynamically generated virtual graphs on top of a relational database.

## Results

### From physical anastylosis empirical predicates to operational research decision making

In this section, the detailing of the operational research model (section “Operational research”) will be used as a guideline to proceed from the asynchronous outputs of the methodological facets (physical anastylosis, reverse engineering and spatio-temporal annotation) for the formulation and validation of the hybrid hypothesis of reconstruction (Fig. 7). We formulate the arch reconstruction problem as a linear programming model, an adaptation of the assignment problem linking voussoir elements to their corresponding slot locations. The reconstruction problem can be formulated as follows: let a collapsed arch be composed of a set of voussoir elements as $C=\{c_1,\ldots ,c_{n}\}$, where $n\in {\mathbb {N}}$ is the number of stones of our problem. Each stone is supposed to be allocated to a location. We denote $E=\{e_1,\ldots ,e_{m}\}$ the set of locations, where $m\ge n$, as some stones have been lost, or broken. For each $1\le i \le n$, for each $1\le j \le m$, we denote $x_{i,j}\in \{0,1\}$ the decision variable of assigning the stone $c_i$ to the location $e_j$ ; thus, if the stone $c_i\in C$ is assigned to the location $e_j\in E$, then $x_{i,j}=1$, else $x_{i,j}=0$. A solution of the problem is an injective function $f:C\rightarrow E$ which allocates each stone $c_i\in C$ to a location $e_j\in E$.

$$\begin{aligned} \begin{aligned} \sum _{j\le m} x_{i,j} = 1 \;|\; \forall i \in [\![1,n]\!] \text{| } \sum _{i\le n} x_{i,j} \le 1 \;|\; \forall j \in [\![1,m]\!] \end{aligned} \end{aligned}$$

#### From Reverse-engineering results to LP model objective function

The first step consists in finding a match between the widths of the voussoirs and the location slots. This is the main criterion for the physical anastylosis, which taken alone has a relatively poor performance of exact relocation (Table 1) due to the distribution of voussoir thicknesses (from 10.5 to 28cm with 49% ranged between 14 and 18cm) but for some pieces with remarkable dimensions. Reverse engineering provides improved accuracy and validation of measurements. For the voussoir elements, measurements taken manually on the physical object are confronted once they are twinned with the photogrammetric model. For the slot location, measurements taken manually on the pre-disaster point cloud are confronted against those of the digital model. Therefore, as a logical predicate, a voussoir element should fit as close as possible to the slot location width.

The second step aims at matching the slot locations with the voussoir fall locations with the information extracted by the spatio-temporal annotation of items between 05/07/2019 and 25/07/2019. Due to the participative nature of the corpus and of the subject, i.e. an evolving pile of rubbles, the scenes are incomplete point clouds with heavy occultations. Nevertheless, the fall position of 31 claveaux has been tracked and identified to their respective inventory pallet. Therefore, due to the arch fall behavior, a voussoir location in the arch should be as close as possible to its fall position.

Hence, in the first step we minimize the delta between the voussoir width $l_{c_i}$ and the slot location width $l_{e_j}$ and in the second step, we minimize the distance between the gravity center of the voussoir and that of the location slot using the cathedral point cloud model as reference space. That is, the objective function is:

$$\begin{aligned} \begin{aligned} min \; \sum _{i=1}^n \sum _{j=1}^m \alpha x_{i,j}|l_{e_j}-l_{c_i}|^r + x_{i,j} (d_{i,j})^s \text{ with } r> 0 \text{, } s> 0 \text{ and } \alpha > 0 \end{aligned} \end{aligned}$$

#### From physical anastylosis predicates and data to LP model constraints

The first archaeological predicate is about a special voussoir element: the keystone. Following the archaeological hypothesis regarding the keystone of the transverse arch (N232) could only be located at the peak slot. By a simple assignation, $x_{kl} = 1 \,|\, k\in [\![1,n]\!]$ and $l\in [\![1,m]\!]$ where $c_k$ is the identified keystone and $e_l$ the peak location.

The second predicate concerns the notches feature present on 38% of the studied voussoirs^23^. While the transverse arch was existing, cubic notches were made on the extrados of the voussoirs to receive planks to support the arch formwork. Often complete, they are sometimes shaped astride two blocks which leads the last hypothesis to define two rules: a half-notch must face another one, a full notch must not face a notch. Let $M_i$ be the $2\times 2$ matrix modeling the corner characteristics of the voussoir $c_i$. For $u,v\in \{1,2\}$, we introduce the following notations: $M_i[u,v]=0$ defines the corner (*u*, *v*) as fragmented, $M_i[u,v]=1$ defines the corner as flat, $M_i[u,v]=2$ defines the corner as half-notched, and $M_i[u,v]=3$ defines the corner as full-notched. Two voussoirs placed side by side share two pairs of angles. We introduce an auxiliary function $f:\{0,1,2,3\} \times \{ 0,1,2,3\} \rightarrow \{1,2\}$ formulating both pairing rules. Hence we formulate the notch constraint $\forall i\in [\![1,n]\!], \forall k\in [\![1,n]\!], \forall j\in [\![1,m-1]\!] \text{, } x_{i,j}+x_{k,j+1} \le f(M_i(1,2),M_k(1,1)) \text{, } x_{i,j}+x_{k,j+1} \le f(M_i(2,2),M_k(2,1)).$

The third feature concerns the location of the notched voussoirs. Once the vault ceiling of the nave could be observed, corpus analysis of the transverse arch found that they were present only in the upper third of the arch. Hence we formulate the notched location constraints defining $M_i=\max \{ M_i(1,1);M_i(1,2);M_i(2,1);M_i(2,2);\} \text{, } b_1=\lfloor m/3 \rfloor$ and $b_2= \lfloor 2m/3+1 \rfloor$ for each $i\in [\![1,n]\!]$, $(M_i-1) \times \left( \sum _{j=1}^{b_1} x_{i,j} + \sum _{j=b_2}^{m} x_{i,j}\right) \le 0$.

**Figure 8:**
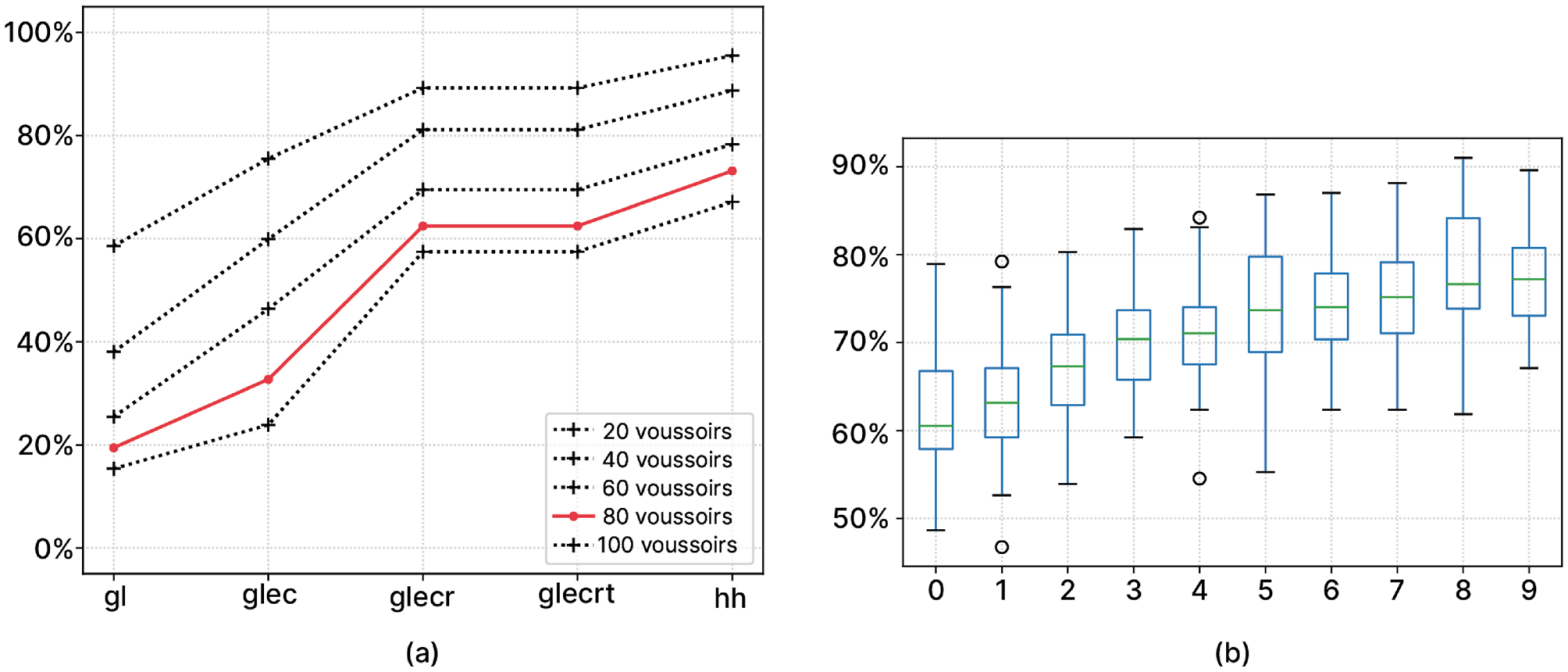
(**a**) Percentage of the milestone models confidence per number of voussoirs; (**b**) percentage of the hybrid hypothesis confidence for 79 voussoirs per number of physical anastylosis provided clusters.

The last predicate concerns the cross lapidary mark signs which are systematically carved on one of the joint faces of each voussoir. These crosses are frequent on the voussoirs of arches from the 1200s but their meaning remains uncertain^41^. The predicate retained was that of a laying sign, intended to indicate the direction in which these blocks were to be placed. Thanks to the rope access technicians who were able to photograph the last voussoir in place to the south of the collapsed transverse arch, the face bearing the cross was oriented towards the keystone of the arch. Finally, the main proficiency issued from physical anastylosis data is to provide several clusters of voussoirs known to be side by side. It is the integration of these clusters and the interplay between the linear programming model, corresponding anastylosis assessment, and the related experts that allows the formulation of the strongest reconstruction hypothesis (Fig. 8b). Let $S \subseteq C$ be a set of $p=card(S)$ voussoirs identified as a cluster. There exists $i_1, ... , i_p \in [\![1,n]\!]$ all different from each other such as $S=\{i_1,...,i_p\}$. Hence we formulate the cluster constraint as : $\forall k\in [\![1,p-1]\!]$, $\forall j\in [\![1,m-1]\!]$, $x_{i_k,j} = x_{i_{k+1},j+1}$.

While data and predicates evolved in the course of the research, we outline 5 milestone models to illustrate the iterative development of the reconstruction hypothesis (Table 1).

**Table 1:** Detailing of the milestone models for reconstruction hypothesis with their performance and uncertainty. With gl: gaussian distribution of width; ec: notches and crosses; r: matching fall location with slot location; t: notches locations; hybrid hypothesis: full LP model with pairs from the physical anastylosis input.

solution type	Width	Fall Location	Keystone	Crosses	Notches	Notches Location	PA Clusters	total number of replaced voussoirs	violations	average model confidence
Physical anastylosis	x		x					36 (50%)	4	na
Digital ‘gl’	x		x					71 (100%)	2	19.46%
Digital ‘glec’	x		x	x	x			71 (100%)	2	32.72%
Digital ‘glecr’	x	x	x	x	x			71 (100%)	0	62.20%
Digital ‘glecrt’	x	x	x	x	x	x		71 (100%)	0	62.44%
Hybrid	x	x	x	x	x	x	x	71 (100%)	0	73.55%

### Performance evaluation of physical anastylosis and operational research hypothesis, performance of hybrid hypothesis

The physical anastylosis located with certainty the keystone and 3 voussoirs^24^, assembled 21 voussoirs as 5 clusters with uncertainty on their selected location, and isolated 11 uncertain voussoir relocation. The evaluation of the physical anastylosis solution via ‘glecrt’ model brought 2 notches constraints violations and 2 notches location constraint violation (Table 1).

Arguably, the more finely the system is characterized by salient criteria, the greater is the confidence in the model and in the derived hybrid hypothesis. Moreover, it seems to stabilize the linear relation linking the confidence per number of voussoirs. All predicates but the notch location provide a steady improvement of about 20% confidence. The most impactful are the width and voussoir fall location objective functions (Fig. 8a). The least impactful is the notch location, as the last hypothesis is weaker than the previous ones for reconstruction. The physical anastylosis-found clusters considered for hybrid hypothesis building provides a steady linear improvement, but does not reduce the substantial standard deviation. We should add here the cascading effect of physical anastylosis cluster identification: the more stone clusters found, the more the solution space is restrained and the higher the corresponding hybrid hypothesis confidence (Fig. 8b). The quantified uncertainty of our model allows us to accurately assess the quality of the hybrid reconstruction hypothesis. The hybrid hypothesis with 5 identified clusters brings a 73.55% confidence in the reconstruction. The 3D visualization of the resulting hypothesis of reconstruction is accessible online at https://reperage.map.cnrs.fr/viewer.

## Discussion

It is a given that digital twins are already identified as an enabling framework beyond any digital technology counterparts. However, few examples of digital twin applications are currently available in the field of cultural heritage. Due to its application scope, the materiality of cultural heritage assets is the favored way to start any scientific approach. The material typology of objects generally conditions the methodology used in their study but in the case of unique assets, the methodology must be tailored to the uniqueness of the case study. How may one reach research reproducibility and reusability in a context where case studies are unique and complex by essence? The common pitfall in the cultural heritage field lies in developing innovative but overfitted methodologies and tools to the assets it studies.

The digital twin experiment as a whole fits the reconstruction question of an arch and is reproducible only for similar questions or assets. Therefore, the reuse of this digital twin experiment appear limited at that aspect, but it is considered as a trade-off in favor of process efficiency. Inversely, because of the process partitioning in facets in the digital twin, all of them are highly reusable by themselves. The autonomy of the facets in the digital twin architecture allows us to decompose and recompose at will depending on the needs of the research question. The versatility of the digital twin framework is a solution for the reuse and the sharing of parts, in opposition to more monolithic architectures.

The overall cohesion happens thanks to horizontal articulation of interdisciplinary perspectives. The multiplicity of viewpoints is both a richness and a challenge at a technical and human level: the collaboration of scientific stakeholders from different backgrounds is mandatory to create innovative solutions for complex problems. Prerequisites for sharing and collaboration—mutual understanding, pedagogical awareness, clarification/mediation and digital tool adoption—lead to strike a balance between actors, physical and digital assets in the digital twin framework.

## Conclusion

The capacity to recover from a traumatic event for a cultural heritage monument is a crucial point for its conservation. This paper showed that an application of the digital twin framework for cultural heritage can solve the related reconstruction questions and support reactive monitoring. This contributed to a dynamic shift from static information management to an adaptive workflow following the information production. It first identified a valid conceptual basis (digital twin) to base upon an iterative modeling method. Then we detailed the harmonization of 4 complementary viewpoints on the reconstruction problem—as a representation, as a hierarchical system, as data provenance, as an optimisation problem—in a hybrid hypothesis. We obtained a quantitative and uncertainty ascertained solution for the reconstruction of the transverse arch of the nave of Notre-Dame de Paris cathedral from its fallen and fragmented components with more than 73% of confidence. With this study, we want to emphasize the importance of flexible digital objects, such as digital twins, less bounded by technologies, to address complex and messy problems in a collaborative environment.

At present, this research of digital twin application is ongoing: it will carry on with the restoration of the cathedral. It is to be expanded to the next phases as it succeeded in the design phase. One future perspective of work is the operating structural monitoring functionality in the digital twin. Future work will concentrate on pattern publishing for knowledge graphs as available semantic bricks for other research projects, automating the spatio-temporal annotation and identification of artifacts in a single processing chain, and moving from one-way links to the digital ecosystem to two-way links.

## Data Availability

The survey data and assessment data described in the current study are available from the corresponding authors on reasonable request. The reconstruction dataset is available at https://github.com/cnrs-mc-umr3495-map/fsp-Reperage. The 3d reconstruction hypothesis is accessible in a 3d viewer at https://reperage.map.cnrs.fr/viewer.
